# Polymorphism detection of *PRKG2* gene and its association with the number of thoracolumbar vertebrae and carcass traits in Dezhou donkey

**DOI:** 10.1186/s12863-022-01101-6

**Published:** 2023-01-04

**Authors:** Tianqi Wang, Ziwen Liu, Xinrui Wang, Yuhua Li, FAHEEM AKHTAR, Mengmeng Li, Zhenwei Zhang, Yandong Zhan, Xiaoyuan Shi, Wei Ren, Bingjian Huang, Changfa Wang, Wenqiong Chai

**Affiliations:** Liaocheng, Research Institute of Donkey High‐Efficiency Breeding and Ecological Feeding, College of Agronomy and Agricultural Engineering, Liaocheng University, Liaocheng, 252059 China

**Keywords:** *PRKG2*, Dezhou donkey, Thoracolumbar vertebrae, Carcass traits, SNPs

## Abstract

**Background:**

Previous studies have shown that the protein kinase cGMP-dependent 2 (*PRKG2*) gene is associated with dwarfism in humans, dogo Argentines, and Angus cattle, as well as with height and osteoblastogenesis in humans. Therefore, the *PRKG2* gene was used as the target gene to explore whether this gene is associated with several thoracolumbar vertebrae and carcass traits in Dezhou donkeys.

**Results:**

In this study, fifteen SNPs were identified by targeted sequencing, all of which were located in introns of the *PRKG2* gene. Association analysis illustrated that the g.162153251 G > A, g.162156524 C > T, g.162158453 C > T and, g.162163775 T > G were significantly different from carcass weight. g.162166224 G > A, g.162166654 T > A, g.162167165 C > A, g.162167314 A > C and, g.162172653 G > C were significantly associated with the number of thoracic vertebrae. g.162140112 A > G was significantly associated with the number and the length of lumbar vertebrae, and g.162163775 T > G was significantly associated with the total number of thoracolumbar vertebrae.

**Conclusion:**

Overall, the results of this study suggest that *PRKG2* gene polymorphism can be used as a molecular marker to breed high-quality Dezhou donkeys.

**Supplementary Information:**

The online version contains supplementary material available at 10.1186/s12863-022-01101-6.

## Introduction

The donkey industry is an integral part of modern animal husbandry, significantly increasing the economic income of both free-range farmers and large farms. Donkey meat is delicious food consumed in some countries, and is highly nutritious and has a unique flavor [1]. Donkeys are uniparous animals and have long growth cycles. Dezhou donkeys reach sexual maturity at about 12–15 months, so molecular breeding of donkeys to improve meat production is necessary and urgent. The number of thoracic vertebrae ranged from 17 to 19, and the number of lumbar vertebrae ranged from 5 to 6 in Dezhou donkey [2]. Previous studies have found that changes in the number of thoracolumbar vertebrae can provide economic benefits. An extra vertebra increases carcass weight by 6 kg [2]. Therefore, it is of great significance to breed multiple thoracolumbar donkeys to improve the quantity of meat.

Many studies have previously demonstrated that variation in the number of thoracolumbar vertebrae can lead to changes in economic traits such as body length and carcass weight in pigs [3] and sheep [4]. In recent years, selection and breeding for multiple thoracolumbar vertebrae traits in pigs, cattle, and sheep have been carried out to analyze the primary loci for thoracolumbar numbers. A point mutation in intron 4 of the *ActRIIB* gene in Small Tailed Han sheep was associated with variation in vertebral number [5]. The *TGFβ3* gene was a candidate gene for the number of vertebrae traits in pigs. The g.105179474 G > A mutation locus on chromosome 7 was associated with the number of ribs and thoracolumbar vertebrae [6]. g.19034 A > C locus of *VRTN* gene can be used as a potential molecular marker for multiple thoracic vertebrae number in Beijing black pigs [7]. However, the selection and breeding for multiple thoracolumbar vertebrae in donkeys have just started. In donkey, the *HOXC8* g.15179224C > T was significantly associated with lumbar vertebrae length (*P* < 0.05), and the g.15179674G > A locus was shown to be significantly associated with the number of lumbar vertebrae (*P* < 0.05) [8]. The *NR6A1* g.18114954C > T is significantly associated with lumber vertebrae number and the total number of thoracolumbar, and individuals with TT genotype had significantly larger value than CC genotype (*P* < 0.05) [2]. Therefore, it is valuable and essential to identify genes affecting multiple thoracolumbar vertebrae numbers and carcass traits in Dezhou donkeys.

**Fig. 1:**
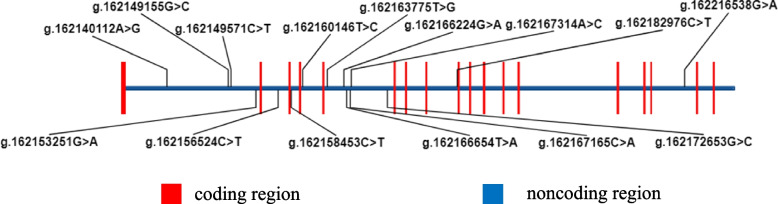
Structure of *PRKG2* gene and locations of fifteen identified *PRKG2* SNPs

Many studies have shown that the *PRKG2* gene was associated with growth traits and skeletal development. *PRKG2* gene is located on chromosome 3 in donkeys and contains eighteen exons and seventeen introns [9] (Fig. 1). Studies have demonstrated that the *PRKG2* gene was associated with dwarfism in American Angus cattle [10], dogo Argentines [11], and humans [12]. The *PRKG2* gene was identified as a candidate gene for human height by genome-wide analysis for copy number variants (CNVs) of 162 patients (149 families) with short stature [13]. The previous studies indicated the *PRKG2* gene as a candidate for osteoblastogenesis [14]. Considering that the *PRKG2* gene affects human height, human height is equivalent to donkey body length, and donkey body length is related to the number of thoracolumbar vertebrae, so the *PRKG2* gene assumes to be associated with the number of thoracolumbar vertebrae and carcass traits. However, the association of the *PRKG2* gene with the number of thoracolumbar vertebrae and carcass traits in Dezhou donkeys has not been reported.

In the present research, genetic variation in the *PRKG2* gene of the Dezhou donkey has been studied using targeted sequencing technology. The targeted sequencing method is a technique to achieve accurate genotype detection by high-depth resequencing of target genes, which has the advantages of high stability, tolerance to sequence conservation and GC content, and can achieve excellent capture efficiency with flexible marker types and capture types [15]. Currently, targeted sequencing technology is widely used in human [16], plant [17], and animal [18]. The study aimed to investigate the genetic variation of the *PRKG2* gene and its correlation with number of thoracolumbar vertebrae and carcass traits in Dezhou donkeys, and provide a specific theoretical basis for molecular breeding of Dezhou donkeys.

## Materials and methods

### Ethics statement

The experimental animals and methods used in this study were approved by the Animal Policy and Welfare Committee of Liaocheng University (No. LC2019-1). The care and use of laboratory animals fully comply with local animal welfare laws, guidelines and policies.

### Animals and phenotypes

Blood samples and trait data were collected from 406 2-year-old Dezhou donkeys at a slaughterhouse in Dezhou, Shandong Province. The 406 Dezhou donkeys in this study were all males and had the same feeding environment. Blood samples were collected from the jugular vein of donkeys using EDTA blood collection tubes and stored in a -20 °C refrigerator immediately. The relevant body size traits of donkeys were measured and recorded. Body height, body length, and chest circumference were measured under the National Standard of the People's Republic of China, "Dezhou Donkey." Carcass weight, the number of lumbar vertebrae, the number of thoracic vertebrae, the length of lumbar vertebrae, the length of thoracic vertebrae, the total number of thoracic and lumbar vertebrae were measured after humanely slaughtered. Carcass traits and the number of thoracolumbar vertebrae data were collected according to the method of Liu et al. (2022). All measurements are performed by the same operator to reduce human error. Table S1 is a summarizes the number of thoracolumbar vertebrae and carcass traits of 406 donkeys. Table S2 shows the mean overall situation of donkeys' thoracolumbar number and carcass traits, and the value is Means ± SE.

### DNA extraction

Genomic DNA was extracted from blood samples using the TIANamp Blood DNA Kit (Tiangen, Beijing, China). After extraction, genomic DNA concentration was measured using a spectrophotometer (B500, Metash, China); a working solution was prepared and adjusted to 30 ng/µL. The samples were placed in a − 20 °C refrigerator for later use.

### SNP detection and genotyping

The 406 genomic DNA samples were sent to Molbreeding Biotechnology Co., Ltd. (Shijiangzhuang, China) for genotyping of the *PRKG2* gene by Targeted Sequencing.

A total of 1292 probes were used in the targeted sequencing, covering 92.39% of the *PRKG2* gene with reference sequence of the donkey *PRKG2* gene (assembly ASM1607732v2; NC_052179; GCA_016077325.2). SNPs with genotype frequencies less than 5% in targeted sequencing results were removed.

### SNPs validation

Sanger sequencing was used to verify the results of targeted sequencing. SNPs located at genomic position 162,150,000–162,160,000 bp in chromosome 3 were randomly selected for validation by Sanger sequencing, and the mutation sites in this region included g.162153251 G > A, g.162156524 C > T, and g.162158453 C > T. Three pairs of primers were designed to amplify three selected SNPs (g.162153251 G > A, g.162156524 C > T, g.162158453 C > T) in the *PRKG2* gene using Primer Premier 5.0 software (Table 1). The PCR amplification was performed in a total of 25 μL reaction, 12.5 μL 2 × Taq PCR Master Mix (Mei5bio, Beijing, China), 8.5 μL ddH_2_O, upstream primer 1 μL, downstream primer 1 μL and DNA template 2 μL were included (Jin et al., 2019).

**Table 1:** Primer sequences, annealing temperature, and products size for Dezhou donkey *PRKG2* gene

Primers/loci	Sequence 5′–3′	Annealing temperature (°C)	Products size (bp)
g.162153251G > A	F:GCACCAGGATACAGACA	62-52touchdown	418
	R:CATAAACTGCCCTCACT		
g.162156524C > T	F:TGTTAGGATACAGCGAGAA	62-52touchdown	818
	R:CCACGATGGCAGAAACT		
g.162158453C > T	F:CTACAACAATGCCCTCA	62-52touchdown	972
	R:TGCTTACCACCTACCTC		

The cycling parameters were as follows: pre-denaturation at 96 ℃ for 5 min, denaturation at 96 ℃ for 20 s, annealing at 62 ℃ for 30 s, and extension at 72 ℃ for 30 s. Each subsequent cycle is reduced by 1 ℃ until 52 ℃, for 10 cycles. 20 s of denaturation at 96 ℃, 30 s of annealing at 52 ℃, and 30 s of stretching at 72 ℃, 35 cycles. 10 min of extension at 72 ℃. 4 ℃ of storage. The specificity of the PCR products was detected using a 2% agarose gel, and samples that were detected for specificity and correct product size were sent to BGI Genomics Co., Ltd (Shanghai, China) for Sanger sequencing, and the results were analyzed using Chromas software (Version V2.6.5, Technelysium Pty Ltd., Queensland, Australia).

### Statistical analyses

Genotype frequencies, allelic frequencies, and the Hardy–Weinberg equilibrium (HWE) were examined using Excel. Population genetic parameters, including homozygosity (Ho), heterozygosity (He), effective allele number (Ne) and the polymorphism information content (PIC) were analyzed using online software (http://www.msrcall.com/, accessed on 24 March 2022) [19]. The association of fifteen SNPs and haplotype combinations of the *PRKG2* gene with the thoracolumbar number and carcass traits was analyzed using a general linear model of SPSS 26.0 (IBM Statistics, Armonk, NY, USA). The results were expressed as means ± SD [20]. Association of fifteen SNPs and haplotype combinations with several thoracolumbar numbers and carcass traits in Dezhou donkeys using a general linear model:

$$Y\mathrm{ij }= \mu + ai + eij$$

where *Y* is the individual phenotypic measurements, *µ* represents the mean for each trait, *a* represents the fixed factor genotype, *e* represents the random error. Least squares means with standard errors were used for the different genotypes and for the number of thoracolumbar vertebrae as well as the carcass traits. Multiple comparisons of the associations were based on Bonferroni-corrected *p*-Values. The different genotypes were considered as fixed effects, the random error as a random effect and the number of thoracolumbar vertebrae and carcass traits as the dependent variable [21]. Linkage disequilibrium (LD) and haplotype construction were performed using Haploview 4.1[22], and haplotypes with frequencies greater than 0.05 were constructed.

## Result

### SNPs identification and genotyping

Targeted sequencing results showed that a total of 485 SNPs were identified (Table S3). Among them, 11 SNPs were located in exons, 457 SNPs were located in introns, 17 SNPs were located downstream of *PRKG2* gene. However, 470 SNPs had a genotype frequency of less than 5%, therefore statistics will not been applied to these data. The locations of these fifteen SNPs are shown schematically in Fig. 1. These fifteen SNPs of *PRKG2* gene were genotyped using sequencing, which generated three genotypes for all locus. The genotyping results of fifteen SNPs of *PRKG2* gene are shown in Table S4. The Sanger sequencing results of the three SNPs (g.162153251 G > A, g.162156524 C > T and g.162158453 C > T) were consistent with the targeted sequencing results. Three samples were randomly selected at three sites from 406 Dezhou donkey DNA samples were randomly selected as the amplification template for three SNPs, and the amplification products were added into 1% agarose gel for electrophoresis identification. Electrophoresis results showed that the bands were single, clear and bright, in line with the expected fragment size.

### Genetic parameter analysis

The genotype and allele frequency were calculated (Table 2). The mutant allele frequency of g. 162,140,112 A > G was the highest, and the normal allele frequency of g. 162,153,251 G > A was the highest. g.162153251 G > A, g.162156524 C > T and g.162216538 G > A were not in HWE. The values of Ho for the fifteen SNPs ranged from 0.2705 to 0.7333, He for the fifteen SNPs ranged from 0.2667 to 0.7295, and Ne for the fifteen SNPs ranged from 1.3636 to 3.6966. Only g.162153251 G > A was in low polymorphism (PIC < 0.25), while the other mutation sites were in moderate polymorphism (g.162149155 G > C, g.162149571 C > T, g.162156524 C > T, g.162158453 C > T, g.162160146 T > C, g.162216538 G > A) (0.25 < PIC < 0.50) and high polymorphism (g.162140112 A > G, g.162163775 T > G, g.162166224 G > A, g.162166654 T > A, g.162167165 C > A, g.162167314 A > C, g.162172653 G > C, g.162182976 C > T) (PIC > 0.50). These data indicate that the genetic diversity of the *PRKG2* gene is relatively high in this population of Dezhou donkeys.

**Table 2:** Genetic parameters of fifteen SNPs in the *PRKG2* gene in Dezhou donkey

	Genotypic frequencies			Allelic frequencies		HWE	Ho	He	Ne	PIC
	DD	ID	II	D	I					
g.162140112A > G	0.5259	0.4000	0.0741	0.7259	0.2741	0.9160	0.2705	0.7295	3.6966	0.7054
g.162149155G > C	0.3990	0.4507	0.1502	0.6244	0.3756	0.4313	0.5310	0.4690	1.8834	0.3590
g.162149571C > T	0.3768	0.4704	0.1527	0.6121	0.3879	0.8506	0.5251	0.4749	1.9043	0.3621
g.162153251G > A	0.0785	0.1599	0.7616	0.1584	0.8416	0.0000	0.7333	0.2667	1.3636	0.2311
g.162156524C > T	0.0630	0.2598	0.6772	0.1929	0.8071	0.0012	0.6886	0.3114	1.4522	0.2629
g.162158453C > T	0.0542	0.3424	0.6034	0.2254	0.7746	0.6951	0.6508	0.3492	1.5365	0.2882
g.162160146 T > C	0.0640	0.3596	0.5764	0.2438	0.7562	0.6167	0.6313	0.3687	1.5841	0.3007
g.162163775 T > G	0.1141	0.4541	0.4318	0.3412	0.6588	0.8395	0.2769	0.7230	3.6112	0.6914
g.162166224G > A	0.1404	0.4901	0.3695	0.3855	0.6145	0.4859	0.2841	0.7159	3.5198	0.6792
g.162166654 T > A	0.1404	0.4901	0.3695	0.3855	0.6145	0.4859	0.2841	0.7159	3.5198	0.6792
g.162167165C > A	0.1404	0.4901	0.3695	0.3855	0.6145	0.4859	0.2841	0.7159	3.5198	0.6792
g.162167314A > C	0.1404	0.4901	0.3695	0.3855	0.6145	0.4859	0.2841	0.7159	3.5198	0.6792
g.162172653G > C	0.1379	0.4926	0.3695	0.3842	0.6158	0.4084	0.2852	0.7148	3.5065	0.6779
g.162182976C > T	0.0815	0.4370	0.4815	0.3000	0.7000	0.4143	0.2781	0.7219	3.5956	0.6935
g.162216538G > A	0.4693	0.3464	0.1844	0.6425	0.3575	0.0000	0.5406	0.4594	1.8498	0.3539

*HWE* Hardy–Weinberg equilibrium, *Ho* homozygosity, *He* heterozygosity, *Ne* effective allele numbers, *PIC* polymorphic information content

PIC < 0.25, low polymorphism; 0.25 < PIC < 0.5, intermediate polymorphism; PIC > 0.5, high polymorphism

II = normal genotype; DD = mutation genotype; ID = heterozygote genotype

### Linkage disequilibrium analysis and haplotype construction

**Fig. 2:**
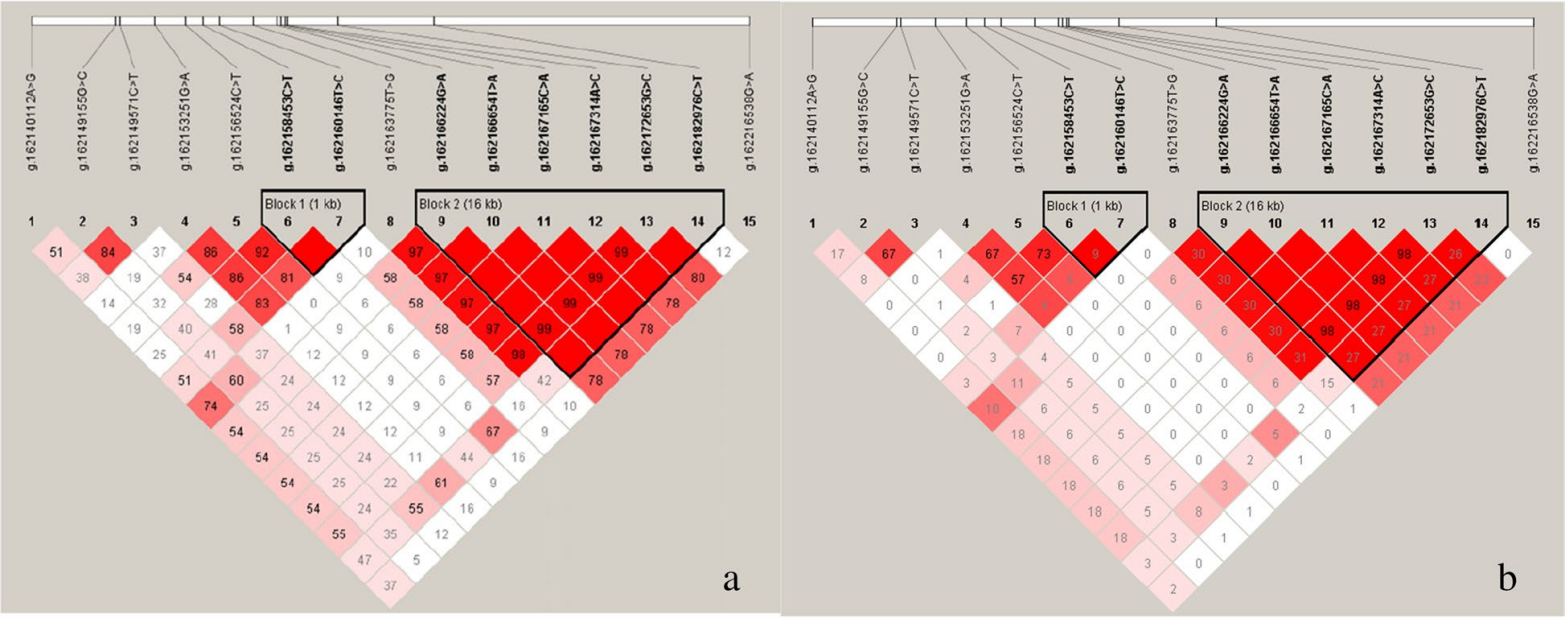
Linkage disequilibrium analysis of fifteen SNPs in Dezhou donkeys. The a-plot is the D' value, and the b-plot is the r^2^ value

Linkage disequilibrium (LD) analysis of the remaining loci showed a strong association between every two SNPs (r^2^ > 0.33) (Fig. 2). Block 1 consisted of two SNPs (g.162158453 C > T, g.162160146 T > C). In block 1, the linkage disequilibrium of g.162158453 C > T with g.162160146 T > C was not very strong (r^2^ < 0.33). Block 2 consisted of six SNPs (g.162166224 G > A, g.162166654 T > A, g.162167165 C > A, g.162167314 A > C, g.162172653 G > C and g.162182976 C > T). In block 2, the linkage disequilibrium of g.162182976 C > T with the other five SNPs (g.162166224 G > A, g.162166654 T > A, g.162167165 C > A, g.162167314 A > C, g.162172653 G > C) was not very strong (r^2^ < 0.33).

 In total, nine haplotypes were constructed. The haplotypes of the *PRKG2* gene and their frequencies in the Dezhou donkey are shown in Table 3. The frequencies of Hap1(CTAAACCC), Hap2(CTGTCAGT), Hap3(CCGTCAGC), Hap4(TTGTCAGC), Hap5(CTGTCAGC), Hap6 (CCAAACCC), Hap7(CCGTCAGT), Hap8(TTAAACCC) and Hap9(TTGTCAGT) were 0.2039, 0.1595, 0.0766, 0.0707, 0.1667, 0.0937, 0.0732, 0.0864, and 0.0675, respectively. Hap1 has the highest frequency, and Hap9 has the lowest frequency. A total of 34 haplotype combinations were found in our population, of which Hap2Hap9(3), Hap3Hap3(5), Hap3Hap6(3), Hap4Hap4(3), Hap4Hap9(3), Hap5Hap5(2), Hap6Hap7(5), Hap6Hap8(3), Hap7Hap7(3), Hap7Hap9(1), Hap8Hap8(5), Hap8Hap9(2) had less than 6 individuals and therefore were not used for association analysis. Hap2Hap6, Hap2Hap8, Hap4Hap6, Hap4Hap7, Hap5Hap6, Hap5Hap7, Hap5Hap8, Hap5Hap9, Hap6Hap6, Hap7Hap8 and Hap9Hap9 combinations were not found in our population.

**Table 3:** Haplotypes of *PRKG2* gene and their frequencies in Dezhou donkey

g.162158453C > T	g.162160146 T > C	g.162166224G > A	g.162166654 T > A	g.162167165C > A	g.162167314A > C	g.162172653G > C	g.162182976C > T	Frequency
C	T	A	A	A	C	C	C	0.2039
C	T	G	T	C	A	G	T	0.1595
C	C	G	T	C	A	G	C	0.0766
T	T	G	T	C	A	G	C	0.0707
C	T	G	T	C	A	G	C	0.1667
C	C	A	A	A	C	C	C	0.0937
C	C	G	T	C	A	G	T	0.0732
T	T	A	A	A	C	C	C	0.0864
T	T	G	T	C	A	G	T	0.0675

### Association analysis of *PRKG2* SNPs with the number of thoracolumbar vertebrae and carcass traits in Dezhou donkeys

The association analysis of *PRKG2* SNPs with the number of thoracolumbar vertebrae and carcass traits in Dezhou donkeys are shown in Table 4. The results of association analysis showed that the g.162149155 G > C and g.162158453 C > T mutations of the *PRKG2* gene were significantly associated with the body height (*P* < 0.05). The g.162140112 A > G was significantly associated with differences in the number and length of lumbar vertebrae (*P* < 0.05). g.162153251 G > A (*P* < 0.01), g.162156524 C > T (*P* < 0.01), g.162158453 C > T (*P* < 0.05) and g.162163775 T > G (*P* < 0.05) were significantly associated with carcass weight. In addition to being significantly associated with body height and carcass weight, g.162158453 C > T was significantly associated with chest circumference (*P* < 0.05). Our analysis showed that there were significant relationships between the different locus of the g.162166224 G > A, g.162166654 T > A, g.162167165 C > A, g.162167314 A > C, g.162172653 G > C and the number of thoracic vertebrae in Dezhou donkey (*P* < 0.05). The g.162163775 T > G locus was significantly associated with the total number of thoracic and lumber, and the total number of thoracolumbar vertebrae was higher in donkeys with the TG genotype than in those with the TT genotype (*P* < 0.01).

**Table 4:** Association of different genotypes of SNPs in *PRKG2* gene with number of thoracolumbar vertebrae and carcass traits in Dezhou donkey. Values with different letters (a > b; A > B) within the same row denote significance levels of *P* < 0.05 and *P* < 0.01, respectively

Loci	Genotype/sample	Body height	Body length	Chest circumference	Carcass weight	Number of lumbar vertebrae	Length of lumbar vertebrae	Number of thoracic vertebrae	Length of thoracic vertebrae	Total number of thoracic and lumbar vertebrae
		(cm)	(cm)	(cm)	(kg)		(cm)		(cm)	
g.162140112A > G	AA/30	135.60 ± 4.28	133.05 ± 5.01	146.25 ± 4.93	154.85 ± 17.05	5.23 ± 0.43ab	24.43 ± 2.82ab	17.83 ± 0.38	73.08 ± 3.56	23.07 ± 0.25
	AG/162	134.87 ± 5.24	132.68 ± 5.84	145.08 ± 5.17	152.27 ± 14.91	5.14 ± 0.34b	23.78 ± 1.80b	17.91 ± 0.33	72.93 ± 3.56	23.04 ± 0.32
	GG/213	134.72 ± 5.02	132.39 ± 6.58	144.60 ± 5.20	150.42 ± 22.25	5.26 ± 0.44a	24.31 ± 2.24a	17.83 ± 0.42	72.73 ± 3.68	23.09 ± 0.37
	*P*-value	0.667	0.816	0.227	0.401	0.011	0.042	0.117	0.809	0.442
g.162149155G > C	GG/61	134.07 ± 44.52ab	132.10 ± 6.30	144.93 ± 4.74	151.92 ± 15.18	5.21 ± 0.41	24.28 ± 2.34	17.85 ± 0.36	72.65 ± 3.42	23.07 ± 0.31
	GC/183	135.62 ± 5.05a	133.03 ± 6.12	145.53 ± 5.14	152.72 ± 19.84	5.17 ± 0.38	23.95 ± 2.11	17.89 ± 0.35	73.07 ± 3.55	23.06 ± 0.33
	CC/162	134.26 ± 5.13b	132.16 ± 6.20	144.24 ± 5.30	149.94 ± 19.89	5.25 ± 0.44	24.24 ± 2.10	17.83 ± 0.43	72.62 ± 3.77	23.08 ± 0.37
	*P*-value	0.018	0.359	0.071	0.399	0.208	0.369	0.373	0.466	0.861
g.162149571C > T	CC/62	134.02 ± 4.87	131.94 ± 6.11	145.11 ± 4.91	152.31 ± 15.61	5.23 ± 0.42	24.34 ± 2.46	17.84 ± 0.37	72.58 ± 3.18	23.06 ± 0.31
	CT/191	135.52 ± 5.19	133.34 ± 6.37	145.27 ± 5.41	152.86 ± 20.29	5.16 ± 0.37	23.97 ± 2.04	17.89 ± 0.35	73.17 ± 3.81	23.05 ± 0.32
	TT/153	134.33 ± 4.85	131.80 ± 5.88	144.41 ± 4.96	149.44 ± 19.15	5.27 ± 0.44	24.20 ± 2.12	17.82 ± 0.43	72.50 ± 3.52	23.09 ± 0.39
	*P*-value	0.035	0.051	0.295	0.244	0.056	0.402	0.257	0.201	0.575
g.162153251G > A	GG/262	134.66 ± 4.92	132.41 ± 6.12	144.80 ± 5.31	151.98 ± 18.75A	5.21 ± 0.41	24.09 ± 2.13	17.85 ± 0.38	72.73 ± 3.52	23.06 ± 0.32
	GA/55	135.01 ± 4.25	132.27 ± 5.68	145.23 ± 4.08	152.63 ± 13.26A	5.20 ± 0.40	24.09 ± 1.97	17.85 ± 0.41	72.85 ± 3.43	23.05 ± 0.30
	AA/27	133.26 ± 6.22	130.72 ± 6.55	142.61 ± 5.59	138.59 ± 31.64B	5.30 ± 0.47	23.98 ± 2.53	17.81 ± 0.48	71.48 ± 3.46	23.11 ± 0.58
	*P*-value	0.300	0.392	0.079	0.002	0.559	0.970	0.893	0.190	0.754
g.162156524C > T	CC/258	134.70 ± 4.96	132.35 ± 6.15	144.64 ± 5.20	151.98 ± 15.99A	5.20 ± 0.40	24.08 ± 2.07	17.85 ± 0.38	72.75 ± 3.59	23.05 ± 0.33
	CT/99	135.37 ± 5.10	132.87 ± 6.56	145.47 ± 4.92	152.67 ± 22.27A	5.21 ± 0.41	24.19 ± 2.23	17.87 ± 0.40	73.27** ± **3.83	23.08 ± 0.34
	TT/24	133.21 ± 5.90	131.02 ± 5.13	144.02 ± 5.31	139.00 ± 31.88B	5.29 ± 0.46	23.98 ± 2.59	17.83 ± 0.48	71.69 ± 3.04	23.13 ± 0.54
	*P*-value	0.155	0.414	0.288	0.005	0.585	0.877	0.902	0.140	0.556
g.162158453C > T	CC/245	134.72 ± 4.79ab	132.41 ± 6.08	144.70 ± 5.13ab	152.03 ± 15.69a	5.22 ± 0.41	24.10 ± 2.08	17.85 ± 0.38	72.75 ± 3.58	23.07 ± 0.34
	CT/139	135.43 ± 5.16a	133.14 ± 6.37	145.69 ± 5.02a	152.12 ± 24.65a	5.19 ± 0.40	24.16 ± 2.20	17.86 ± 0.39	73.14 ± 3.78	23.06 ± 0.31
	TT/22	132.45 ± 6.43b	130.30 ± 5.65	142.5 ± 5.85b	141.48 ± 13.08b	5.27 ± 0.46	23.93 ± 2.50	17.91 ± 0.43	71.73 ± 2.85	23.18 ± 0.50
	*P*-value	0.030	0.116	0.015	0.042	0.680	0.891	0.761	0.205	0.281
g.162160146 T > C	TT/234	135.06 ± 5.30	132.76 ± 6.49	144.32 ± 3.67	150.90 ± 12.56	5.38 ± 0.50	24.15 ± 2.29	17.87 ± 0.38	72.99 ± 3.75	23.08 ± 0.39
	TC/146	134.64 ± 4.78	132.33 ± 5.72	144.49 ± 5.05	151.54 ± 20.67	5.16 ± 0.37	23.96 ± 1.82	17.86 ± 0.36	72.63 ± 3.48	23.03 ± 0.35
	CC/26	134.00 ± 4.01	131.81 ± 5.84	145.26 ± 5.37	151.52 ± 18.98	5.22 ± 0.42	24.65 ± 2.40	17.69 ± 0.47	72.54 ± 3.26	23.09 ± 0.33
	*P*-value	0.495	0.665	0.308	0.987	0.034	0.315	0.075	0.591	0.185
g.162163775 T > G	TT/174	134.72 ± 4.78	132.41 ± 5.95	144.72 ± 5.13	150.97 ± 19.52ab	5.17 ± 0.38	23.94 ± 2.08	17.84 ± 0.38	72.84 ± 3.41	23.02 ± 0.31B
	TG/183	135.03 ± 5.30	133.01 ± 6.25	145.30 ± 5.02	153.70 ± 16.01a	5.22 ± 0.42	24.20 ± 2.19	17.91 ± 0.36	73.08 ± 3.69	23.13 ± 0.38A
	GG/46	134.57 ± 5.22	131.46 ± 6.76	144.25 ± 5.90	145.12 ± 27.43b	5.28 ± 0.46	24.24 ± 2.14	17.74 ± 0.44	71.92 ± 4.01	23.02 ± 0.26AB
	*P*-value	0.786	0.284	0.362	0.022	0.206	0.453	0.021	0.155	0.005
g.162166224G > A	GG/150	134.84 ± 4.84	132.31 ± 6.47	144.80 ± 5.53	152.55 ± 16.03	5.25 ± 0.43	24.23 ± 2.06	17.80 ± 0.43b	72.52 ± 3.82	23.05 ± 0.37
	GA/199	134.73 ± 5.32	132.48 ± 6.22	145.08 ± 4.78	152.29 ± 15.10	5.18 ± 0.39	23.99 ± 2.17	17.90 ± 0.33a	73.07 ± 3.38	23.09 ± 0.35
	AA/57	135.25 ± 4.64	133.36 ± 5.21	144.72 ± 5.56	150.03 ± 24.56	5.23 ± 0.42	24.22 ± 2.28	17.84 ± 0.41ab	72.78 ± 3.88	23.07 ± 0.26
	*P*-value	0.797	0.543	0.844	0.503	0.315	0.535	0.040	0.375	0.583
g.162166654 T > A	TT/150	134.84 ± 4.84	132.31 ± 6.47	144.8 ± 5.53	150.03 ± 24.56	5.25 ± 0.43	24.23 ± 2.06	17.80 ± 0.43b	72.52 ± 3.82	23.05 ± 0.37
	TA/199	134.73 ± 5.32	132.48 ± 6.22	145.08 ± 4.78	152.29 ± 15.10	5.18 ± 0.39	23.99 ± 2.17	17.90 ± 0.33a	73.07 ± 3.38	23.09 ± 0.35
	AA/57	135.25 ± 4.64	133.36 ± 5.21	144.72 ± 5.56	152.55 ± 16.03	5.23 ± 0.42	24.22 ± 2.28	17.84 ± 0.41ab	72.78 ± 3.88	23.07 ± 0.26
	*P*-value	0.797	0.543	0.844	0.503	0.315	0.535	0.040	0.375	0.583
g.162167165C > A	CC/150	135.25 ± 4.64	132.31 ± 6.47	144.80 ± 5.53	150.03 ± 24.56	5.23 ± 0.42	24.23 ± 2.06	17.80 ± 0.43b	72.52 ± 3.82	23.05 ± 0.37
	CA/199	134.73 ± 5.32	132.48 ± 6.22	145.08 ± 4.78	152.29 ± 15.10	5.18 ± 0.39	23.99 ± 2.16	17.90 ± 0.33a	73.07 ± 3.38	23.09 ± 0.35
	AA/57	134.84 ± 4.84	133.36 ± 5.21	144.72 ± 5.56	152.55 ± 16.03	5.25 ± 0.43	24.22 ± 2.28	17.84 ± 0.41ab	72.78 ± 3.88	23.07 ± 0.26
	*P*-value	0.797	0.543	0.844	0.503	0.315	0.535	0.040	0.375	0.583
g.162167314A > C	AA/150	134.84 ± 4.84	132.31 ± 6.47	144.80 ± 5.53	150.03 ± 24.56	5.25 ± 0.43	24.23 ± 2.06	17.80 ± 0.43b	72.52 ± 3.82	23.05 ± 0.37
	AC/199	134.73 ± 5.32	132.48 ± 6.22	145.08 ± 4.78	152.29 ± 15.10	5.18 ± 0.39	23.99 ± 2.17	17.90 ± 0.33a	73.07 ± 3.38	23.09 ± 0.35
	CC/57	135.25 ± 4.64	133.36 ± 5.21	144.72 ± 5.56	152.55 ± 16.03	5.23 ± 0.42	24.22 ± 2.28	17.84 ± 0.41ab	72.78 ± 3.88	23.07 ± 026
	*P*-value	0.797	0.543	0.844	0.503	0.315	0.535	0.040	0.375	0.583
g.162172653G > C	GG/150	134.81 ± 4.85	132.24 ± 6.46	144.73 ± 5.42	149.77 ± 24.36	5.25 ± 0.43	24.23 ± 2.06	17.80 ± 0.43b	72.52 ± 3.83	23.05 ± 0.37
	GC/200	134.71 ± 5.33	132.52 ± 6.22	145.08 ± 4.92	152.38 ± 15.33	5.18 ± 0.39	23.98 ± 2.17	17.90 ± 0.33a	73.07 ± 3.36	23.08 ± 0.34
	CC/56	135.39 ± 4.54	133.44 ± 5.22	144.91 ± 5.42	152.92 ± 15.93	5.23 ± 0.43	24.26 ± 2.28	17.84 ± 0.42ab	72.79 ± 3.91	23.07 ± 0.26
	*P*-value	0.671	0.466	0.821	0.379	0.296	0.475	0.037	0.367	0.588
g.162182976C > T	CC/195	134.65 ± 5.24	132.33 ± 6.20	144.64 ± 5.36	151.93 ± 15.65	5.22 ± 0.41	24.20 ± 2.24	17.87 ± 0.39	72.77 ± 3.58	23.09 ± 0.33
	CT/177	134.88 ± 4.72	132.82 ± 6.11	145.38 ± 4.89	151.32 ± 23.04	5.18 ± 0.38	23.91 ± 2.02	17.85 ± 0.36	72.93 ± 3.51	23.03 ± 0.33
	TT/33	135.77 ± 5.66	132.48 ± 6.57	144.06 ± 5.44	149.86 ± 16.55	5.36 ± 0.49	24.61 ± 2.08	17.82 ± 0.47	72.72 ± 4.47	23.18 ± 0.47
	*P*-value	0.493	0.743	0.240	0.840	0.049	0.158	0.728	0.893	0.038
g.162216538G > A	GG/66	134.38 ± 4.59	131.76 ± 6.59	144.42 ± 5.26	151.79 ± 17.39	5.21 ± 0.41	24.03 ± 2.05	17.79 ± 0.41	72.08 ± 3.71b	23.00 ± 0.39
	GA/124	134.59 ± 5.51	131.69 ± 6.06	144.72 ± 5.21	151.83 ± 14.82	5.20 ± 0.40	24.14 ± 2.07	17.84 ± 0.41	72.57 ± 3.40ab	23.04 ± 0.35
	AA/168	135.20 ± 4.51	133.24 ± 5.57	145.12 ± 5.22	151.78 ± 20.14	5.19 ± 0.39	24.05 ± 2.09	17.90 ± 0.36	73.31 ± 3.59a	23.09 ± 0.31
	*P*-value	0.406	0.530	0.614	1.000	0.927	0.887	0.115	0.037	0.157

Values with different letters (a > b; A > B) within the same row denote significance levels of *P* < 0.05 and *P* < 0.01, respectively

### Association analysis of *PRKG2* haplotype combinations with the number of thoracolumbar vertebrae and carcass traits in Dezhou donkeys

Different haplotype combinations were not significantly associated with body height, body length, chest circumference, carcass weight, the number of lumbar vertebrae, the length of lumbar vertebrae, the number of thoracic vertebrae, the length of thoracic vertebrae, the total number of thoracic and lumbar vertebrae (*P* > 0.05) (Table S5). The number of lumbar vertebrae of haplotype combination Hap4Hap8(5.56 ± 0.53) donkeys was 0.56 higher than that of haplotype combination Hap6Hap9(5.00 ± 0.00) donkeys with the lowest number of lumbar vertebrae. The lumbar length of haplotype combination Hap4Hap8(25.44 ± 2.92) donkeys was 2.15 cm longer than the haplotype combination Hap6Hap9(23.29 ± 1.91) donkeys with the shortest lumbar length. The total number of thoracolumbar vertebrae of haplotype combination Hap4Hap8(23.44 ± 0.73) donkeys was 0.53 higher than that of haplotype combination Hap3Hap8 (22.91 ± 0.30) donkeys with the lowest the total number of thoracolumbar vertebrae. Carcass weight of haplotype combination Hap3Hap5(157.92 ± 20.43) donkeys was 26.42 kg higher than that of haplotype combination Hap3Hap9 (131.50 ± 59.98) donkeys with the lowest carcass weight. The number of thoracic vertebrae of haplotype combination Hap3Hap5(18.17 ± 0.41) donkeys was 0.47 higher than that of haplotype combination Hap3Hap7(17.70 ± 0.48) donkeys with the lowest number of thoracic vertebrae. The thoracic length of haplotype combination Hap3Hap5(75.50 ± 2.51) donkeys was 4.82 cm longer than the haplotype combination Hap2Hap7(70.68 ± 3.95) donkeys with the shortest thoracic distance.

## Discussion

The Dezhou donkey is one of China's five best donkey breeds, with high production characteristics and stable genetic performance [23]. In recent decades, breeding efforts have focused on animals that meet people's basic needs, such as pigs and chickens. After satisfying food and clothing, people's demand for food began to pursue nutrition and health. Many studies showed that donkey meat is of great nutritional value [24]. However, as a special-type economic animal, the progress of donkey breeding is slow. Therefore, the identification of molecular markers affecting economic traits is essential to accelerate the molecular breeding process of Dezhou donkeys.

Fifteen SNPs were identified in the *PRKG2* gene of the Dezhou donkey for the first time in this study, and SNPs located in the *PRKG2* gene have not been previously reported in donkeys. Polymorphisms in the *PRKG2* gene have also been found in humans, dogs, and cattle. The mutation c.1705 C > T found in the exonic region of the human genome is associated with acral dysplasia [25]. Koltesa et al. (2009) found that the C/T transition in exon 15 of the American Angus cattle *PRKG2* gene introduced a stop codon (R678X) and demonstrated that the R678X resulted in the loss of regulation of *COL2* and *COL10* mRNA expression. R678X is a pathogenic mutation in American Angus cattle dwarfism. The fifteen SNPs we identified were all located in the intron region. Similarly, the c.1634 + 1 G > T locus found in the intron region of dogo Argentines is a candidate pathogenic variant of dwarfism. Radiographs of dogs with dwarfism show reduced levels of endochondral ossification in epiphyseal plates and premature closure of the distal ulna epiphysis line [11]. Currently, genetic variants of the *PRKG2* gene have not been identified in horses, sheep, and pigs.

In the fifteen SNPs confirmed, only one mutant site was low polymorphic, six mutant sites were moderately polymorphic, and eight mutant sites were highly polymorphic. This result indicates a relatively high level of polymorphism in this population. However, considering that our group consisted entirely of two-year-old male donkeys, our results have some limitations. The g.162153251 G > A, g.162156524 C > T, and g.162216538 G > A locus are not in HWE, indicating that they may be affected by artificial selection, natural selection, migration, and population size, and the genetics of these three sites are unstable [26]. The average observed heterozygosity of fifteen SNPs was 0.3575, and the average expected heterozygosity was 0.6022, this suggests that the Dezhou donkey population is rich in genetic variation [27].

Growth traits are important indicators of breeding, and thirteen SNPs were significantly associated with the thoracolumbar number and carcass traits. Unfortunately, g.162160146 T > C and g.162182976 C > T were not associated with all traits; this may be due to the small sample size used in our study [19, 21]. g.162149155 G > C and g.162158453 C > T were significantly associated with the body height of the Dezhou donkey (*P* < 0.05). Duyvenvoorde et al. (2014) showed that the *PRKG2* gene was identified as a candidate gene for human height. However, fifteen SNPs of the *PRKG2* gene were not significantly associated with body length in our study. g.162140112 A > G was significantly associated with lumbar spine number and length (*P* < 0.05). g.162166224 G > A, g.162166654 T > A, g.162167165 C > A, g.162167314 A > C and g.162172653 G > C were significantly associated with the number of thoracic vertebrae (*P* < 0.05), and g.162163775 T > G was significantly associated with the total number of thoracolumbar vertebrae (*P* < 0.01). Yi et al. (2021) found that the *PRKG2* gene promotes adipogenesis and impairs osteoblastogenesis. It is the opposite of our results, g.162140112 A > G, g.162163775 T > G, g.162166224 G > A, g.162166654 T > A, g.162167165 C > A, g.162167314 A > C and g.162172653 G > C may affect the function of osteoclastogenesis in the *PRKG2* gene has been hypothesized, but the mechanisms involved need to be further investigated.

Haplotype combinations are highly likely to be inherited together [26]. Although SNP sites were significantly associated with carcass traits and the number of thoracolumbar vertebrae, association analysis revealed that the constructed haplotype combinations were not significantly associated with the number of thoracolumbar vertebrae and carcass traits. A possible explanation for this is that haplotype combination with the highest value of traits had a sample size of less than 6 were not included in the association analysis of this study [28]. Furthermore, donkeys with haplotype combination Hap4Hap8 had the significant length of lumbar vertebrae, number of lumbar vertebrae, and the total number of thoracolumbar vertebrae compared to donkeys with other haplotype combinations. Donkeys with haplotype combination Hap3Hap5 had the greatest carcass weight, length of thoracic vertebrae, and the number of thoracic vertebrae compared to donkeys with other haplotype combinations. Although there were no significant differences between haplotype combinations and traits, the dominant haplotype combinations Hap4Hap8 and Hap3Hap5 that we found were able to bring about some positive effects.

Similarly, SNPs located in introns significantly associated with growth performance compared with SNPs located in exons and non-coding regions. For example, a novel g.3624 A > G polymorphism in intron 2 of the *TBX3* gene is significantly associated with body size in donkeys [20]. Numerous studies have shown that SNPs located in introns are associated with alternative splicing. Alternative splicing plays a vital role in regulating biological functions [29]. The g.19970 A > G site found in intron 11 of the cow *INCNEP* gene enhances the action of the splicing factor SRSF1, SRSF1(IgM-BRCA1), and SRSF5. It changes the binding sites of splicing factor SRSF6, generating a new transcript that alters gene expression [30]. g.11043 C > T in the intron 1 of the *SPEF2* gene that alters the binding of the splicing factor binding protein SC35 to the target sequence, and it was hypothesized that this mutation is essential for the production of new transcripts and therefore has an effect on bull semen trait production [31]. The fifteen SNPs that were newly identified by us affected the shear factor binding sites that need to be further confirmed.

## Conclusions

In this study, we focused on the variation of the *PRKG2* gene and its association with the number of thoracolumbar vertebrae and carcass traits of donkeys. Based on the targeted and Sanger sequencing methods, we found fifteen SNPs of the *PRKG2* gene, all located in the intron region. The results showed that the *PRKG2* gene could be a molecular marker with multiple thoracolumbar vertebrae and better carcass traits in donkeys, laying the foundation for breeding high-quality donkey breeds with high meat production.

## Supplementary Information

**Additional file 1.**

## Data Availability

Genotyping results have been submitted to the Sequence Read Archive (SRA), study accession number: PRJNA884985. The data is accessible at the following link: https://www.ncbi.nlm.nih.gov/bioproject/PRJNA884985. Additional data generated during this study are included in this published article. Data are also available upon request from the authors.
